# Dietary intake in the Personalized Medicine Research Project: a resource for studies of gene-diet interaction

**DOI:** 10.1186/1475-2891-10-13

**Published:** 2011-01-28

**Authors:** Lacie Strobush, Richard Berg, Deanna Cross, Wendy Foth, Terrie Kitchner, Laura Coleman, Catherine A McCarty

**Affiliations:** 1 Center for Human Genetics, Marshfield Clinic Research Foundation, 1000 N. Oak Ave (MLR), Marshfield, WI 54449, USA; 2 Biomedical Informatics Research Center, Marshfield Clinic Research Foundation, 1000 N. Oak Ave (ML8), Marshfield, WI 54449, USA; 3 Epidemiology Research Center, Marshfield Clinic Research Foundation, 1000 N. Oak Ave (ML2), Marshfield, WI 54449, USA

## Abstract

**Background:**

To describe the dietary intake of participants in the Personalized Medicine Research Project (PMRP), and to quantify differences in nutrient intake by smoking status and APOE4-a genetic marker that has been shown to modify the association between risk factors and outcomes.

**Methods:**

The PMRP is a population-based DNA, plasma and serum biobank of more than 20,000 adults aged 18 years and older in central Wisconsin. A questionnaire at enrollment captures demographic information as well as self-reported smoking and alcohol intake. The protocol was amended to include the collection of dietary intake and physical activity via self-reported questionnaires: the National Cancer Institute 124-item Diet History Questionnaire and the Baecke Physical Activity Questionnaire. These questionnaires were mailed out to previously enrolled participants. APOE was genotyped in all subjects.

**Results:**

The response rate to the mailed questionnaires was 68.2% for subjects who could still be contacted (alive with known address). Participants ranged in age from 18 to 98 years (mean 54.7) and 61% were female. Dietary intake is variable when comparing gender, age, smoking, and APOE4. Over 50% of females are dietary supplement users; females have higher supplement intake than males, but both have increasing supplement use as age increases. Food energy, total fat, cholesterol, protein, and alcohol intake decreases as both males and females age. Female smokers had higher macronutrient intake, whereas male nonsmokers had higher macronutrient intake. Nonsmokers in both genders use more supplements. In females, nonsmokers and smokers with APOE4 had higher supplement use. In males, nonsmokers with APOE4 had higher supplement use between ages 18-39 only, and lower supplement use at ages above 39. Male smokers with APOE4 had lower supplement use.

**Conclusion:**

Dietary intake in PMRP subjects is relatively consistent with data from the National Health and Nutrition Examination Survey (NHANES). Findings suggest a possible correlation between the use of supplements and APOE4. The PMRP dietary data can benefit studies of gene-environment interactions and the development of common diseases.

## Background

With the completion of the Human Genome Project, the laboratory tools to quantify genetic variation in human populations exist. Analyzing genetic variation could lead to the discovery of genetic predictors of disease. In addition to those predictors, it is important to quantify gene-environment interactions that modify genetic associations. Dietary intake is associated with multiple health outcomes and is one of the critical, potentially modifiable, environmental exposures to consider in gene-environment studies [1]. Food frequency questionnaires (FFQ) are the most cost-effective tool to measure usual dietary intake in large cohort studies, but caution must be taken with the interpretation and use of macronutrient data from FFQ [1]. Interactions involving alcohol intake as an environmental factor have been studied to illustrate its impact on development of certain health outcomes [2]. Another common, modifiable, environmental risk factor for consideration in gene-environment studies is smoking; dietary intake has been shown to vary by smoking status [3].

Apolipoprotein E (APOE) is one of the most commonly researched genes in studies of gene-environment interactions. Through its function as a ligand and its involvement with chylomicrons, very-low density lipoproteins (VLDL), and high-density lipoproteins (HDLs), APOE helps maintain cholesterol and fat levels in the body [4]. The APOE gene has three alleles, one of which is E4. The E4 allele has been associated with both coronary heart disease (CHD) and early onset of Alzheimer's disease [5]. Total cholesterol and LDL cholesterol levels in general are highest in people who have an E4 allele [6,7]. Some studies have suggested that APOE4 carriers who are smokers are at increased risk for coronary heart disease compared to non-smokers [5].

The Personalized Medicine Research Project (PMRP) is a population-based DNA, plasma and serum biobank designed to facilitate genetic epidemiology and pharmacogenetic studies [8-11]. The comprehensive medical record of the Marshfield Clinic is ideal for the identification of affected cases and appropriate controls; however, limited information about personal exposure is collected in a standardized fashion in the context of routine clinical care. Therefore, assessments of known, potentially modifiable, risk factors for disease were included in the study protocol. They include smoking status, alcohol intake, and a detailed FFQ and physical activity questionnaire. The purpose of this paper is to describe the PMRP biobank as a resource for gene-diet studies, to quantify the extent to which smoking status, alcohol consumption, and the APOE genotype are associated with dietary intake in the population, and to explain how these factors may need to be considered as co-variants in future gene-nutrient studies.

## Methods

### Personalized Medicine Research Project (PMRP)

Details of the PMRP have been published previously [8-11]. In summary, the project was designed to establish a large biobank consisting of DNA, plasma and serum from a large representative sample. Since central Wisconsin has a relatively stable population and the majority of residents receive care at a Marshfield Clinic, the geographic area is ideal for research over a long period of time. Participants that were invited were residents of at least 18 years of age, living in one of 19 zip-codes surrounding Marshfield, WI, and the vast majority received most of their medical care in the Marshfield Clinic system. After subjects have signed the written consent form, which allows access to their comprehensive Marshfield Clinic medical record, subjects complete a brief questionnaire about demographics, smoking status, alcohol intake, and health history. DNA, plasma, and serum samples were extracted and stored from whole blood. To extract the DNA, the Gentra's AUTOPURE^® ^system was used. White blood cells were isolated and lysed; through multiple steps of centrifugation and decanting, DNA was obtained, washed and stored at -80°C [8]. All procedures were reviewed and approved by the Marshfield Clinic Institutional Review Board.

### Quantification of dietary intake

The study protocol was amended after nearly 18,000 subjects were enrolled in PMRP to include usual dietary intake and physical activity. Usual dietary intake was measured using the validated National Cancer Institute 124-item Diet History Questionnaire (DHQ) [12-17]. For those subjects already enrolled, the DHQ was mailed out, with a second mailing and follow-up phone calls as needed to increase the participation rate. The completed questionnaires were scanned and nutrient files were created using the software package Diet*Calc (http://riskfactor.cancer.gov/DHQ/dietcalc/). Questionnaires with more than half of the pages or items not complete were excluded from analysis. Standard units were used. ATE CSFII refers to the units for vitamin E. CSFII stands for Continuing Survey of Food Intakes by Individuals; ATE stands for alpha-tocopherol equivalent, which is a form of vitamin E absorbed by humans. IU and RE refer to the units for measuring vitamin A. IU stands for international units, and RE stands for retinol equivalents.

### Quantification of APOE4 genotype

Matrix-assisted laser desorption/ionization time-of-flight (MALDI-TOF) mass spectrometry was used to genotype the polymorphisms of the APOE gene. PCR reactions, which used primers designed by the Assay Designer 2.05-software from Sequenom, amplified designated regions of DNA. Primer extension reactions were performed to generate allele-specific products that are one base longer than the original primer. The products were placed onto a matrix arrayed silicon chip and analyzed by a MALDI-TOF mass spectrometer and Sequenom SpectroTYPER 3.4 software. The mass spectrometer determines alleles based on different molecular weights [11].

### Statistical analyses

Preliminary analyses included tabular and graphical summaries describing participant demographics, dietary intake, and APOE genotypes. In the primary study analyses we analyze dietary intake for associations with body size (BMI), smoking status and APOE4, while controlling for expected age and gender differences. Stratification and graphical displays were used to investigate the plausibility and consistency of potential associations, and to identify potential interactions and confounding. Group comparisons of dietary intake stratified by gender and age were conducted using rank-based methods (Wilcoxon and Kruskal-Wallis tests). Analyses were conducted using SAS^® ^(version 9.2, SAS Inc., Cary, NC). Results were considered statistically significant at the 5% level (p < 0.05) without adjustment for multiple comparisons.

## Results

**Figure 1:**
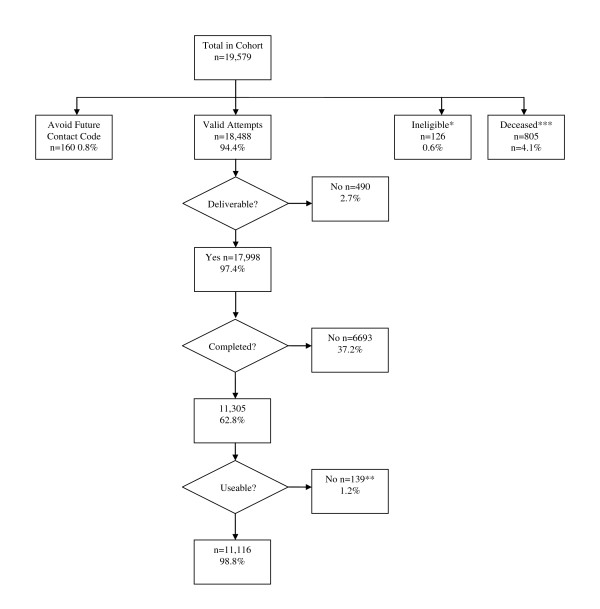
**Diagram of DHQ mailing for PMRP dietary cohort as of September 16, 2009**. The diagram includes participants enrolled on or before 2/3/09. *nursing home and non-English speaking, **too many missing fields on the DHQ

The response rate to the mailed questionnaires was 62.8% for subjects who could still be contacted (alive with known address). Approximately 3% of participants could not be located and 4% were deceased. Figure 1 illustrates the tracking of questionnaires mailed out to participants. The 11,166 with dietary information ranged in age from 18 to 98 years (mean 54.9 years) and 6821 (61%) were female. Demographic characteristics of the dietary cohort and a comparison with non-responders are summarized in Table 1. Responders were more likely to be female, older, and never smokers.

**Table 1:** Descriptive characteristics of the dietary cohort and comparison with non-responders

	**Males**	**Females**	**Overall**	**Non-responders**
Subjects (n %)	4,345 39%	6,821 61%	11,166	8,413 52% Female
Median age (yr)	57.1	53.4	54.9	46.4
Minimum	18	18	18	18
Maximum	96	98	98	103
Diabetes (n %)	769 18%	904 13%	1673 15%	1250 15%
Smoking history (n %)				
Never	2024 47%	4211 62%	6235 56%	4298 51%
Current	655 15%	983 14%	1638 15%	2065 25%
Other or unknown	1666 38%	1627 24%	3293 29%	2050 24%
E4 genotype (n %)	1198 28%	1750 26%	2948 26%	2271 27%
Median BMI (kg/m^2^)	28.7	27.8	28.2	27.8
Minimum	14.7	15.0	14.7	14.9
Maximum	71.1	69.9	71.1	66.4
Median HDL (mg/dL)	43.0	53.5	49.0	46.0
Minimum	16.0	15.0	15.0	11.0
Maximum	135.0	147.0	147.0	141.0

Dietary intake varied by age, gender, smoking and the E4 allele. Trends seen within data are statistically significant, unless otherwise noted. Table 2 compares the percent use of various supplements between females and males of different age groups. The percent use of supplements for females increases as age group increases. Over fifty percent of women in each age group consume supplements; similar trends are seen for males. When comparing males and females in the same age group, the percent-use of the various supplements is lower in males than in females. Vitamin C supplements are consumed most frequently by both females and males.

**Table 2:** Percent use of supplements by gender and age in the Personalized Medicine Research Project

**Females**				**Males**			
Age Group	18-39	40-59	60+	Age Group	18-39	40-59	60+
N*	1644	2645	2512	N*	824	1620	1900
	% use	% use	% use		% use	% use	% use
Vit. A IU	59.6	65.1	71.2	Vit. A IU	38.3	47.3	56.6
Vit. A RAE	59.6	65.1	71.2	Vit. A RAE	38.3	47.3	56.6
Beta Car.	59.0	64.7	70.7	Beta Car.	37.9	47.1	56.6
Vit. E	60.3	68.9	75.6	Vit. E	38.8	49.4	60.4
Vit. C	65.2	72.5	76.6	Vit. C	44.7	53.4	62.5
Thiamin	59.5	66.6	71.6	Thiamin	38.0	47.7	57.4
Riboflavin	59.5	66.6	71.6	Riboflavin	38.0	47.7	57.4
Niacin	59.7	67.0	72.1	Niacin	38.2	48.3	58.6
Vit. B6	59.8	67.4	72.5	Vit. B6	38.3	48.0	58.3
Folic Acid	60.2	65.3	71.7	Folic Acid	38.1	47.3	57.9
Vit. B12	58.7	64.2	70.0	Vit. B12	37.6	46.7	56.1
Calcium	27.8	55.0	69.7	Calcium	13.5	17.3	30.9
Magnesium	54.1	60.0	61.1	Magnesium	32.9	42.3	48.8
Iron	56.5	61.9	63.4	Iron	33.7	42.7	50.8
Zinc	54.8	61.2	62.7	Zinc	34.0	43.3	50.5
Copper	54.1	60.0	61.1	Copper	32.9	42.3	48.8
Vit. D	58.7	64.2	70.0	Vit. D	37.6	46.7	56.1
Selenium	0.5	1.6	2.1	Selenium	0.2	2.2	5.1

*N refers to the total number of females or males within the particular age group

Tables 3 and 4 compare the dietary intake between different age groups of females (Tables 3) and males (Table 4). Supplement-use summaries for the subset who use supplements are listed at the bottom of each table. The results suggest that with increasing age in females, food energy, total fat, cholesterol, protein, and alcohol intake decreases. Conversely, as women age, the average supplement intake increased. Similar trends were observed in males, regarding the mean and median intake of food energy, total fat, cholesterol, protein, alcohol, and supplement intake.

**Table 3:** Dietary intake in females by age in the Personalized Medicine Research Project

**Age Group**	**18-39**			**40-59**			**60+**			
	N*	Mean	Median	N*	Mean	Median	N*	Mean	Median	K-W Test p-value
Food energy (kcal)	2147	1859.5	1637.4	2752	1674.7	1522.7	1997	1415.5	1341.1	< 0.001
Total fat (g)	2147	65.2	56.1	2752	61.6	53.8	1997	51.3	46.0	< 0.001
Cholesterol (mg)	2147	199.3	171.4	2752	185.0	161.7	1997	154.3	137.3	< 0.001
Protein (g)	2147	71.6	64.2	2752	67.3	61.7	1997	54.5	50.8	< 0.001
Alcohol (g)	2147	6.6	2.1	2752	5.9	1.4	1997	3.1	0.6	< 0.001
Vitamin A (IU CSFII)	2147	8958.8	6375.6	2752	9921.8	7354.3	1997	8549.4	6695.0	< 0.001
Vitamin A (mcg RE CSFII)	2147	1264.8	1003.5	2752	1324.5	1096.7	1997	1157.6	999.3	< 0.001
Vitamin E (mg ATE CSFII)	2147	8.3	6.9	2752	8.4	7.3	1997	7.5	6.6	< 0.001
Vitamin C (mg)	2147	134.6	101.3	2752	127.1	104.3	1997	124.5	108.2	< 0.065
Zinc (mg)	2147	11.0	9.8	2752	10.4	9.5	1997	8.7	8.1	< 0.001
Selenium (mcg)	2147	86.2	76.4	2752	81.2	74.0	1997	67.0	62.1	< 0.001
Total Vitamin A Activity (mcg)	2147	838.4	719.7	2752	828.2	719.9	1997	721.8	659.8	< 0.001
Beta Carotene	2147	3198.6	2055.7	2752	3657.3	2524.0	1997	3101.7	2276.5	< 0.001
Lutein|Zeaxanthin (mcg NDS)	2147	2444.7	1643.3	2752	2467.9	1727.9	1997	1998.6	1474.5	< 0.001
Lycopene (mcg NDS R)	2147	7726.0	5611.2	2752	6815.3	4998.7	1997	6096.7	4022.5	< 0.001
Beta Carotene Equivalents (mcg)	2147	3701.3	2373.0	2752	4237.7	2899.1	1997	3599.2	2632.4	< 0.001
Vitamin E Total	2147	7.8	6.4	2752	7.9	6.7	1997	6.7	5.9	< 0.001
Supp. Vitamin A (IU)	2147	2094.6	331.8	2752	2928.0	3571.4	1997	3496.9	5000.0	< 0.001
Supp. Vitamin A (mcg RAE)	2147	628.4	99.6	2752	878.4	1071.4	1997	1049.1	1500.0	< 0.001
Supp. Beta Carotene	2147	246.8	39.8	2752	339.6	428.6	1997	410.7	600.0	< 0.001
Supp. Vitamin E	2147	16.8	4.4	2752	58.5	20.1	1997	78.0	20.1	< 0.001
Supp. Vitamin C	2147	81.1	17.1	2752	150.7	60.0	1997	176.8	60.0	< 0.001
Supp. Zinc	2147	6.1	1.0	2752	8.6	10.7	1997	9.2	15.0	< 0.001
Supp. Selenium	2147	0.3	0.0	2752	0.8	0.0	1997	0.8	0.0	< 0.001
**Supplement Users Only**										
Supp. Vitamin A (IU)	992	3497.2	5000.0	1722	4182.1	5000.0	1789	4843.7	5000.0	< 0.001
Supp. Vitamin A (mcg RAE)	992	1049.2	1500.0	1722	1254.6	1500.0	1789	1453.1	1500.0	< 0.001
Supp. Beta Carotene	982	410.2	600.0	1712	492.9	600.0	1775	572.1	600.0	< 0.001
Supp. Vitamin E	1003	24.6	20.1	1823	74.1	20.1	1900	100.0	20.1	< 0.001
Supp. Vitamin C	1085	115.5	60.0	1917	188.4	60.0	1924	229.1	60.0	< 0.001
Supp. Zinc (mg)	900	1.4	2.0	1587	1.6	2.0	1535	1.8	2.0	< 0.001
Supp. Selenium (mcg)	9	42.9	42.9	43	42.9	42.9	53	42.9	42.9	1.000

*N refers to the total number of participants per age group

**Table 4:** Dietary intake in males by age in the Personalized Medicine Research Project

**Age Group**		**18-39**			**40-59**			**60+**		
	N*	Mean	Median	N*	Mean	Median	N*	Mean	Median	K-W Test p-value
Total fat (g)	1105	99.2	85.9	1764	87.0	74.7	1515	69.6	61.9	< 0.001
Cholesterol (mg)	1105	304.0	258.1	1764	263.1	226.1	1515	214.9	186.8	< 0.001
Protein (g)	1105	106.0	91.1	1764	91.7	81.3	1515	71.2	64.4	< 0.001
Alcohol (g)	1105	21.4	5.1	1764	15.5	3.5	1515	10.2	1.8	< 0.001
Vitamin A (IU CSFII)	1105	9292.7	6715.6	1764	9490.8	7456.3	1515	8731.1	6842.3	< 0.001
Vitamin A (mcg RE CSFII)	1105	1442.2	1152.8	1764	1384.7	1201.5	1515	1270.6	1086.9	< 0.001
Vitamin E (mg ATE CSFII)	1105	10.9	8.8	1764	10.2	8.7	1515	9.0	7.8	< 0.001
Vitamin C (mg)	1105	157.3	113.0	1764	137.4	111.4	1515	125.8	107.5	< 0.034
Zinc (mg)	1105	16.3	14.0	1764	14.3	12.6	1515	11.6	10.3	< 0.001
Selenium (mcg)	1105	130.9	114.0	1764	116.1	102.4	1515	92.1	83.5	< 0.001
Total Vitamin A Activity (mcg)	1105	1048.6	862.2	1764	944.1	838.1	1515	848.5	733.7	< 0.001
Beta Carotene	1105	3111.2	1971.9	1764	3290.3	2335.0	1515	3035.9	2195.7	< 0.001
Lutein|Zeaxanthin (mcg NDS)	1105	2640.6	1740.4	1764	2368.8	1773.8	1515	2140.5	1544.8	< 0.001
Lycopene (mcg NDS R)	1105	10491	7941.1	1764	9438.5	6621.3	1515	7770.7	5163.8	< 0.001
Beta Carotene Equivalents (mcg)	1105	3571.6	2266.4	1764	3818.0	2697.1	1515	3521.6	2538.3	< 0.001
Vitamin E Total	1105	10.0	8.1	1764	9.3	8.0	1515	7.9	6.8	< 0.001
Supp. Vitamin A (IU)	1105	1222.9	0.0	1764	2258.9	82.1	1515	2806.4	3571.4	< 0.001
Supp. Vitamin A (mcg RAE)	1105	366.9	0.0	1764	677.7	24.6	1515	841.9	1071.4	< 0.001
Supp. Beta Carotene	1105	144.1	0.0	1764	264.9	9.9	1515	327.5	428.6	< 0.001
Supp. Vitamin E	1105	11.6	0.0	1764	35.9	2.4	1515	50.5	20.1	< 0.001
Supp. Vitamin C	1105	54.8	0.0	1764	106.3	17.1	1515	130.8	60.0	< 0.001
Supp. Zinc	1105	3.8	0.0	1764	6.6	0.0	1515	7.8	0.0	< 0.001
Supp. Selenium	1105	0.2	0.0	1764	1.2	0.0	1515	2.2	0.0	< 0.001
**Supplement Users Only**										
Supp. Vitamin A (IU)	316	3059.8	3571.4	766	4229.3	5000.0	1075	4930.3	5000.0	< 0.001
Supp. Vitamin A (mcg RAE)	316	917.9	1071.4	766	1268.8	1500.0	1075	1479.1	1500.0	< 0.001
Supp. Beta Carotene	312	360.5	428.6	763	494.1	600.0	1076	579.4	600.0	< 0.001
Supp. Vitamin E	320	22.9	14.4	801	61.6	20.1	1147	83.4	20.1	< 0.001
Supp. Vitamin C	368	115.6	60.0	865	173.2	60.0	1187	212.1	60.0	< 0.001
Supp. Zinc (mg)	271	1.2	1.4	685	1.6	2.0	927	1.9	2.0	< 0.001
Supp. Selenium (mcg)	2	42.9	42.9	36	42.9	42.9	96	42.9	42.9	1.000

*N refers to the total number of participants per age group

Tables 5, 6 and 7 illustrate supplement use stratified by age group, gender, smoking, and E4 allele status. Comparing females on smoking status alone, the nonsmokers generally consume more dietary supplements throughout all age groups. This similar trend is seen in males as well. The data show, once again, that females had more supplement use percentages than males when comparing relative age groups. Differences in supplement use are seen between those that have the E4 allele and those that do not. In the nonsmoking females, those with the E4 allele had higher supplement intake than nonsmokers without E4. Current smoking females with E4 have a higher supplement intake than those without E4. This trend is relatively consistent throughout all age groups in females. The data show some inconsistencies between males and females. In nonsmoking males between ages 18-39, those with E4 have higher percent use than those without E4; in the same age group, smokers without the E4 allele had higher use of supplements. In the other two age groups, nonsmoking males with E4 have lower percent use of supplements than those without E4. Current smoking males with E4 had a lower percent use than those without E4. This trend is seen in current smokers among each age group in males. Supplement use by E4 differed between genders. In general, females with E4 had higher supplement use percentages, and males with E4 had lower supplement use percentages compared to those without E4.

**Table 5:** Percent use of supplements by gender, smoking status, and E4 in subjects aged 18-39 in the Personalized Medicine Research Project

	**Never Smoked**		**Current Smokers**	
	**With E4**	**No E4**	**With E4**	**No E4**
**Females**				
N*	293	775	104	296
	% Use	% Use	% Use	% Use
Vit. A IU	68.9	61.4	49.0	48.0
Vit. A RAE	68.9	61.4	49.0	48.0
Beta Car.	68.3	60.5	49.0	48.3
Vit. E	69.6	61.9	51.0	49.0
Vit. C	73.0	66.7	52.9	53.7
Thiamin	68.3	60.9	49.0	48.6
Riboflavin	68.3	60.9	49.0	48.6
Niacin	68.3	61.4	49.0	48.6
Vit. B6	68.3	61.4	49.0	49.0
Folic Acid	67.9	62.1	50.0	49.0
Vit. B12	67.9	60.1	48.1	48.0
Calcium	32.4	25.0	27.9	25.0
Magnesium	62.1	55.4	44.2	44.9
Iron	63.1	57.5	45.2	49.0
Zinc	63.1	56.0	45.2	45.6
Copper	62.1	55.4	44.2	44.9
Vit. D	67.9	60.1	48.1	48.0
Selenium	0.0	1.0	0.0	0.3
**Males**				
N*	125	377	59	156
	% Use	% Use	% Use	% Use
Vit. A IU	40.8	39.5	30.5	35.9
Vit. A RAE	40.8	39.5	30.5	35.9
Beta Car.	42.4	38.7	28.8	35.3
Vit. E	40.8	39.5	33.9	37.2
Vit. C	46.4	43.8	40.7	46.8
Thiamin	40.8	38.7	30.5	35.9
Riboflavin	40.8	38.7	30.5	35.9
Niacin	41.6	38.7	32.2	35.9
Vit. B6	40.8	38.7	32.2	36.5
Folic Acid	40.8	38.7	32.2	35.9
Vit. B12	40.8	38.7	28.8	35.3
Calcium	16.0	11.9	15.3	12.8
Magnesium	34.4	33.7	25.4	30.8
Iron	34.4	33.7	30.5	33.3
Zinc	35.2	34.7	30.5	31.4
Copper	34.4	33.7	25.4	30.8
Vit. D	40.8	38.7	28.8	35.3
Selenium	0.0	0.30	1.70	0.0

*N refers to the number of participants

**Table 6:** Percent use of supplements by gender, smoking status, and E4 in subjects aged 40-59 in the Personalized Medicine Research Project

	**Never Smoked**		**Current Smokers**	
	**With E4**	**No E4**	**With E4**	**No E4**
**Females**				
N*	426	1186	105	306
	% Use	% Use	% Use	% Use
Vit. A IU	70.9	64.3	65.7	57.8
Vit. A RAE	70.9	64.3	65.7	57.8
Beta Car.	70.4	63.8	65.7	56.9
Vit. E	74.2	68.0	65.7	61.1
Vit. C	78.4	72.2	71.4	64.1
Thiamin	73.5	65.5	67.6	58.5
Riboflavin	73.5	65.5	67.6	58.5
Niacin	73.9	65.9	67.6	58.5
Vit. B6	73.5	66.6	69.5	59.5
Folic Acid	71.4	64.6	67.6	57.5
Vit. B12	70.2	63.4	65.7	56.9
Calcium	59.9	55.6	42.9	43.8
Magnesium	67.1	58.9	56.2	53.6
Iron	68.3	61.4	57.1	54.9
Zinc	67.8	59.9	57.1	54.2
Copper	67.1	58.9	56.2	53.6
Vit. D	70.2	63.4	65.7	56.9
Selenium	1.9	1.5	1.9	1.0
**Males**				
N*	230	624	91	215
	% Use	% Use	% Use	% Use
Vit. A IU	46.1	47.1	41.8	46.0
Vit. A RAE	46.1	47.1	41.8	46.0
Beta Car.	46.1	47.1	38.5	46.5
Vit. E	47.8	49.5	40.7	47.4
Vit. C	50.9	53.0	49.5	51.6
Thiamin	45.7	47.6	38.5	47.0
Riboflavin	45.7	47.6	38.5	47.0
Niacin	45.7	48.2	38.5	47.9
Vit. B6	46.5	47.6	39.6	47.0
Folic Acid	46.5	47.1	38.5	46.5
Vit. B12	45.7	46.8	37.4	46.0
Calcium	10.4	17.8	13.2	15.3
Magnesium	43.0	41.5	33.0	41.9
Iron	43.0	42.0	34.1	41.9
Zinc	43.9	42.1	35.2	42.8
Copper	43.0	41.5	33.0	41.9
Vit. D	45.7	46.8	37.4	46.0
Selenium	2.2	1.4	2.2	2.8

*N refers to the number of participants

**Table 7:** Percent use of supplements by gender, smoking status, and E4 in subjects aged 60 and older in the Personalized Medicine Research Project

	**Never Smoked**		**Current Smokers**	
	**With E4**	**No E4**	**With E4**	**No E4**
**Females**				
N*	374	1157	39	133
	% Use	% Use	% Use	% Use
Vit. A IU	73.3	71.3	71.8	60.9
Vit. A RAE	73.3	71.3	71.8	60.9
Beta Car.	72.7	70.7	71.8	61.7
Vit. E	78.3	76.2	74.4	66.9
Vit. C	79.9	76.1	74.4	72.2
Thiamin	74.3	71.6	71.8	61.7
Riboflavin	74.3	71.6	71.8	61.7
Niacin	74.3	72.4	71.8	61.7
Vit. B6	74.9	72.5	71.8	62.4
Folic Acid	74.1	71.3	71.8	63.9
Vit. B12	72.5	69.8	71.8	60.2
Calcium	68.2	72.0	56.4	58.6
Magnesium	64.2	60.3	64.1	51.1
Iron	65.8	62.9	64.1	54.1
Zinc	65.0	62.1	66.7	51.9
Copper	64.2	60.3	64.1	51.1
Vit. D	72.5	69.8	71.8	60.2
Selenium	1.6	2.0	0.0	3.8
**Males**				
N*	190	477	33	101
	% Use	% Use	% Use	% Use
Vit. A IU	47.9	57.9	39.4	56.4
Vit. A RAE	47.9	57.9	39.4	56.4
Beta Car.	47.9	58.1	39.4	57.4
Vit. E	53.7	60.6	45.5	57.4
Vit. C	54.2	63.5	48.5	61.4
Thiamin	48.4	59.5	39.4	56.4
Riboflavin	48.4	59.5	39.4	56.4
Niacin	51.1	60.4	39.4	57.4
Vit. B6	48.4	60.6	45.5	57.4
Folic Acid	48.4	58.7	45.5	57.4
Vit. B12	47.4	57.2	39.4	56.4
Calcium	26.8	32.1	33.3	29.7
Magnesium	40.5	50.1	36.4	48.5
Iron	42.1	52.4	36.4	50.5
Zinc	42.1	52.0	39.4	49.5
Copper	40.5	50.1	36.4	48.5
Vit. D	47.4	57.2	39.4	56.4
Selenium	4.7	6.5	3.0	1.0

*N refers to the number of participants

Table 8 compares the dietary intake between smoking and nonsmoking females and males. For females, the data suggest that the dietary intake for food energy, total fat, cholesterol, alcohol, vitamin E (mg ATE CSFII), selenium, and lycopene was higher in smokers versus nonsmokers. Furthermore, the dietary intake for vitamin A (IU CSFII), vitamin A (mcg RE CSFII), and vitamin C was higher in nonsmokers than in smokers. No differences were seen in supplement intake between the two groups. Differences can also be seen between smokers and nonsmokers when comparing 25% and 75% quartile values. The results regarding smoking and dietary intake for males were not statistically significant. Nonsmokers generally consumed healthier diets, as evidenced by using more supplements, consuming higher dietary vitamin C, and consuming less alcohol.

**Table 8:** Dietary intake by gender and smoking status in the Personalized Medicine Research Project. The total number of participants who smoked and never smoked is indicated by "N" beneath the respective category.

**Females**							
	**Never Smoked**			**Smoked 100+**			
N*		4262		2538			
	25%	Median	75%	25%	Median	75%	Wilcoxon p-value
Food energy (kcal)	1092.3	1468.8	1942.1	1146.3	1539.9	2066.2	< 0.001
Total fat (g)	35.7	51.2	72.3	37.6	54.8	77.2	< 0.001
Cholesterol (mg)	102.8	153.8	220.1	109.6	164.2	235.3	< 0.001
Protein (g)	42.4	58.6	78.3	42.9	60.0	81.1	0.056
Alcohol (g)	0.1	1.1	3.3	0.3	1.7	6.3	< 0.001
Vitamin A (IU CSFII)	4394.1	6915.6	11608	4104.1	6639.4	11198	0.002
Vitamin A (mcg RE CSFII)	703.1	1051.3	1563.0	654.6	1005.5	1536.2	0.003
Vitamin E (mg ATE CSFII)	4.7	6.8	9.9	4.8	7.2	10.5	0.007
Vitamin C (mg)	69.7	107.1	158.2	63.2	100.4	157.4	< 0.001
Zinc (mg)	6.6	9.1	12.1	6.6	9.3	12.4	0.089
Selenium (mcg)	50.5	70.2	94.2	52.4	72.9	99.2	0.002
Total Vitamin A Activity (mcg)	479.9	706.7	989.6	451.1	692.1	1000.1	0.206
Beta Carotene	1374.5	2316.2	4265.4	1267.5	2266.6	4176.5	0.060
Lutein|Zeaxanthin (mcg NDS)	1029.3	1636.6	2579.3	1002.5	1615.4	2619.1	0.506
Lycopene (mcg NDS R)	3026.7	4815.3	7901.4	3194.0	5075.2	8470.1	0.003
Beta Carotene Equivalents (mcg)	1586.3	2689.2	4912.3	1468.6	2609.5	4793.5	0.026
Vitamin E Total	4.4	6.3	9.2	4.4	6.5	9.6	0.026
**Males**							
	Never Smoked			Smoked 100+			
N*		2045			2233		
	25%	Median	75%	25%	Median	75%	Wilcoxon p-value
Food energy (kcal)	1499.7	2051.0	2772.5	1426.6	1955.4	2720.8	0.050
Total fat (g)	50.4	73.9	104.4	49.2	71.2	104.9	0.156
Cholesterol (mg)	147.1	222.4	324.5	147.5	216.6	326.0	0.685
Protein (g)	57.6	79.3	110.3	54.1	75.5	105.4	0.001
Alcohol (g)	0.6	2.8	10.0	0.6	3.5	15.2	0.001
Vitamin A (IU CSFII)	4734.1	7164.8	11634	4505.0	6892.0	10910	0.054
Vitamin A (mcg RE CSFII)	777.6	1179.8	1760.9	744.9	1109.3	1652.2	0.002
Vitamin E (mg ATE CSFII)	5.8	8.4	12.1	5.7	8.3	12.2	0.342
Vitamin C (mg)	73.1	113.1	176.0	67.8	107.5	167.6	0.004
Zinc (mg)	8.9	12.2	17.4	8.5	11.8	16.7	0.006
Selenium (mcg)	71.3	98.8	136.8	68.2	96.8	134.2	0.148
Total Vitamin A Activity (mcg)	554.6	834.8	1209.9	523.7	785.4	1154.5	0.001
Beta Carotene	1294.6	2163.3	3954.2	1333.7	2184.9	3822.1	0.813
Lutein|Zeaxanthin (mcg NDS)	1103.4	1737.5	2696.4	1030.2	1633.7	2641.8	0.072
Lycopene (mcg NDS R)	3976.0	6436.8	10382	3846.7	6409.3	10736	0.780
Beta Carotene Equivalents (mcg)	1499.2	2499.0	4593.6	1528.5	2523.0	4431.0	0.705
Vitamin E Total	5.4	7.6	11.1	5.2	7.5	11.1	0.196

*N refers to the number of participants

Tables 8 and 9 illustrate the supplement intake between females (Table 9) and males (Table 10) stratified by having the E4 allele or not. The data suggest that females with the E4 allele have higher supplement intake than those without it; however, when looking at the "supplement users only" data, there is little to no difference by E4 status. As for males, the data suggest that those without the E4 allele have higher supplement intake. With some exceptions, the same general trend is seen within the "supplement users only" data.

**Table 9:** Supplement intake by APOE4 genotype in females in the Personalized Medicine Research Project

	**With E4**				**No E4**				
Nutrient	N*	Mean	S.D.	Median	N*	Mean	S.D.	Median	Wilcoxon p-value
Supp. Vitamin A (IU)	1750	2965.2	2801.5	3571.4	5071	2789.8	2845.7	3571.4	0.006
Supp. Vitamin A (mcg RAE)	1750	889.6	840.4	1071.4	5071	836.9	853.7	1071.4	0.006
Supp. Beta Carotene	1750	350.6	329.0	428.6	5071	325.1	323.0	428.6	0.002
Supp. Vitamin E	1750	52.6	113.6	20.1	5071	50.8	108.8	20.1	0.046
Supp. Vitamin C	1750	140.5	273.5	60.0	5071	134.4	262.3	60.0	0.076
Supp. Thiamin (mg)	1750	1.4	2.0	1.5	5071	1.3	1.9	1.1	0.002
Supp. Riboflavin (mg)	1750	1.3	1.5	1.7	5071	1.2	1.5	1.2	0.002
Supp. Niacin (mg)	1750	13.7	13.4	20.0	5071	12.7	12.9	14.3	0.003
Supp. Vitamin B6 (mg)	1750	4.4	10.6	2.0	5071	4.3	10.8	1.4	0.014
Supp. Folic Acid (mcg)	1750	230.2	191.8	285.7	5071	215.8	194.8	285.7	0.004
Supp. Vitamin B12 (mcg)	1750	3.3	2.8	4.3	5071	3.1	2.8	4.3	0.002
Supp. Vitamin D (mcg)	1750	286.6	365.6	16.6	5071	290.5	371.6	16.6	0.866
Supp. Calcium (mg)	1750	50.8	47.3	71.4	5071	47.1	47.2	28.6	0.003
Supp. Magnesium (mg)	1750	10.4	10.3	12.9	5071	9.9	10.2	12.9	0.111
Supp. Iron (mg)	1750	8.4	8.2	10.7	5071	7.9	8.3	4.3	0.007
Supp. Zinc (mg)	1750	1.0	0.9	1.4	5071	0.9	0.9	0.6	0.003
Supp. Copper (mg)	1750	221.4	186.7	285.7	5071	205.9	188.0	285.7	0.002
Supp. Selenium (mcg)	1750	0.6	4.9	0.0	5071	0.7	5.4	0.0	0.375
**Supplement Users Only**									
Supp. Vitamin A (IU)	1208	4295.6	2377.5	5000.0	3295	4293.5	2450.7	5000.0	0.931
Supp. Vitamin A (mcg RAE)	1208	1288.7	713.3	1500.0	3295	1288.0	735.2	1500.0	0.931
Supp. Beta Carotene	1201	510.9	275.4	600.0	3268	504.4	267.2	600.0	0.558
Supp. Vitamin E	1261	73.1	128.1	20.1	3465	74.3	124.8	20.1	0.468
Supp. Vitamin C	1314	187.2	301.5	60.0	3612	188.6	293.9	60.0	0.262
Supp. Thiamin (mg)	1228	2.0	2.1	1.5	3323	1.9	2.1	1.5	0.841
Supp. Riboflavin (mg)	1228	1.9	1.5	1.7	3323	1.8	1.4	1.7	0.841
Supp. Niacin (mg)	1231	19.5	11.8	20.0	3346	19.2	11.3	20.0	0.857
Supp. Vitamin B6 (mg)	1234	6.2	12.2	2.0	3366	6.5	12.7	2.0	0.634
Supp. Vitamin B12 (mcg)	1220	330.2	140.6	400.0	3310	330.6	142.0	400.0	0.954
Supp. Folic Acid (mcg)	1195	4.9	2.0	6.0	3238	4.8	2.0	6.0	0.697
Supp. Vitamin D (mcg)	941	533.0	342.5	500.0	2727	540.2	349.1	500.0	0.686
Supp. Calcium (mg)	1086	81.9	32.5	100.0	2936	81.4	32.5	100.0	0.681
Supp. Magnesium (mg)	1110	16.4	8.2	18.0	3059	16.5	8.0	18.0	0.290
Supp. Iron (mg)	1107	13.2	6.5	15.0	2998	13.3	6.6	15.0	0.908
Supp. Zinc (mg)	1086	1.6	0.6	2.0	2936	1.6	0.7	2.0	0.681
Supp. Copper (mg)	1195	324.2	133.1	400.0	3238	322.5	133.1	400.0	0.697
Supp. Selenium (mcg)	23	42.9	0.0	42.9	82	42.9	0.0	42.9	1.000

*N refers to the number of participants

**Table 10:** Supplement intake by APOE4 genotype in males in the Personalized Medicine Research Project

	**With E4**				**No E4**				
Nutrient	N	Mean	S.D.	Median	N	Mean	S.D.	Median	Wilcoxon p-value
Supp. Vitamin A (IU)	1198	2068.6	2769.4	0.0	3146	2234.1	2781.4	82.1	0.047
Supp. Vitamin A (mcg RAE)	1198	620.6	830.8	0.0	3146	670.2	834.4	24.6	0.047
Supp. Beta Carotene	1198	239.8	301.6	0.0	3146	262.4	322.6	9.9	0.043
Supp. Vitamin E	1198	34.0	92.0	0.3	3146	35.5	94.2	1.3	0.118
Supp. Vitamin C	1198	95.1	220.8	8.2	3146	105.0	243.7	17.1	0.312
Supp. Thiamin (mg)	1198	0.9	1.5	0.0	3146	0.9	1.6	0.0	0.042
Supp. Riboflavin (mg)	1198	0.8	1.2	0.0	3146	0.9	1.3	0.0	0.042
Supp. Niacin (mg)	1198	9.6	13.0	0.0	3146	10.5	13.8	0.3	0.052
Supp. Vitamin B6 (mg)	1198	2.3	7.4	0.0	3146	2.8	8.4	0.0	0.029
Supp. Folic Acid (mcg)	1198	156.8	189.9	0.0	3146	171.7	194.1	6.6	0.020
Supp. Vitamin B12 (mcg)	1198	2.3	2.8	0.0	3146	2.5	2.8	0.1	0.020
Supp. Vitamin D (mcg)	1198	66.8	199.6	0.0	3146	88.3	228.9	0.0	0.010
Supp. Calcium (mg)	1198	34.2	45.6	0.0	3146	37.4	46.7	0.0	0.029
Supp. Magnesium (mg)	1198	6.7	9.0	0.0	3146	7.4	9.4	0.0	0.022
Supp. Iron (mg)	1198	5.9	8.1	0.0	3146	6.5	8.5	0.0	0.029
Supp. Zinc (mg)	1198	0.7	0.9	0.0	3146	0.7	0.9	0.0	0.029
Supp. Copper (mg)	1198	151.4	185.6	0.0	3146	166.1	189.4	6.6	0.020
Supp. Selenium (mcg)	1198	1.1	6.8	0.0	3146	1.4	7.6	0.0	0.242
**Supplement Users Only**									
Supp. Vitamin A (IU)	568	4362.9	2482.6	5000.0	1589	4423.2	2373.4	5000.0	0.415
Supp. Vitamin A (mcg RAE)	568	1308.9	744.8	1500.0	1589	1327.0	712.0	1500.0	0.415
Supp. Beta Carotene	564	509.4	236.2	600.0	1587	520.2	268.8	600.0	0.653
Supp. Vitamin E	603	67.5	120.6	20.1	1665	67.0	121.0	20.1	0.690
Supp. Vitamin C	652	174.8	275.2	60.0	1768	186.8	300.7	60.0	0.890
Supp. Thiamin (mg)	573	1.8	1.8	1.5	1603	1.8	1.9	1.5	0.363
Supp. Riboflavin (mg)	573	1.7	1.3	1.7	1603	1.8	1.3	1.7	0.363
Supp. Niacin (mg)	583	19.8	12.2	20.0	1628	20.3	12.9	20.0	0.492
Supp. Vitamin B6 (mg)	579	4.8	10.1	2.0	1621	5.4	11.0	2.0	0.217
Supp. Vitamin B12 (mcg)	571	329.0	137.8	400.0	1610	335.6	136.4	400.0	0.244
Supp. Folic Acid (mcg)	558	4.9	2.0	6.0	1574	5.0	1.9	6.0	0.185
Supp. Vitamin D (mcg)	241	331.9	332.1	250.0	738	376.4	339.2	285.7	0.069
Supp. Calcium (mg)	489	83.7	30.8	100.0	1394	84.4	30.9	100.0	0.466
Supp. Magnesium (mg)	503	15.8	6.7	18.0	1431	16.3	7.1	18.0	0.260
Supp. Iron (mg)	505	13.9	6.6	15.0	1437	14.1	7.0	15.0	0.516
Supp. Zinc (mg)	489	1.7	0.6	2.0	1394	1.7	0.6	2.0	0.466
Supp. Copper (mg)	558	325.0	132.2	400.0	1574	331.9	129.1	400.0	0.185
Supp. Selenium (mcg)	31	42.9	0.0	42.9	103	42.9	0.0	42.9	1.000

*N refers to the number of participants

## Discussion

The dietary intake of participants in the Personalized Medicine Research Project (PMRP) is a useful resource to assist in studies regarding gene-diet interactions. Statistically significant findings were seen when analyzing the PMRP dietary data for differences associated with smoking, alcohol consumption, and the APOE genotype.

The National Health and Nutrition Examination Survey (NHANES) is a survey that documents dietary intake on a yearly basis. Comparing the PMRP dietary intake of macronutrients with that of NHANES, the PMRP dietary intake is relatively similar. In ages eighteen and above, percent energy from protein, carbohydrates, total fat, and saturated fat are similar between the PMRP and NHANES. NHANES data revealed slightly higher food energy, cholesterol, natural folate, and sodium intake. PMRP intake was significantly higher for calcium [18]. This finding could be due to the higher consumption of dairy foods and vegetables associated the farming in Wisconsin.

Differences have been seen in dietary intake between smokers and nonsmokers. Interactions between diet and smoking can lead to negative health outcomes. Findings of previous studies suggest that smokers consume less fiber, vegetables, whole grains, fruits but more bacon/luncheon meats, whole milk, and calories in general [3]. Smokers also are less likely than nonsmokers to consume vitamins, minerals and/or supplements [3]. Our results are generally consistent with previous findings. In PMRP, women who smoke have a lower intake of supplements and vitamins, and a higher intake in food energy, fat, cholesterol, and protein. Similarly, supplement intake was lower and alcohol consumption was higher in smoking males.

Studies have shown the APOE gene to be associated with increased risk for coronary heart disease (CHD) and Alzheimer's Disease. Smoking increases the risk for CHD alone, but its interaction with the APOE4 genotype can cause an even higher risk [2]. This demonstrates a possible gene-environment interaction. Our findings suggest that females with the E4 allele have higher supplement intake and smokers with the E4 allele have slightly lower use. Males with the E4 allele have lower supplement intake, but higher use is seen in nonsmokers. These data suggest that people may have started supplement use to prevent diseases for which they have in increased risk (possibly due to family history) and these diseases are associated with APOE. Vitamin E supplementation has been shown to decrease the risk of some diseases and supplements are marketed directly to consumers for this purpose.

One strength of the PMRP dietary intake data is the size of the cohort that the data includes. The relatively high response rate is another strength of the resource. However, there were some response limitations. For early participants, dietary data were collected several years after their initial enrollment. The initial 17,000 participants were enrolled within the first eighteen months after the project began in 2002. The first set of mailings was not sent until 2006. Approximately 4% of participants were deceased by the time the DHQs were mailed. 2.7% of participants were not able to be contacted. Males were less likely to respond to the questionnaire. Although this information should be considered, the percentages are quite low and do not present a strong impact on the collected data.

## Conclusions

Detailed dietary history data are available for more than 11,000 adult participants in a biobank with DNA, plasma and serum samples linked to a comprehensive electronic health record. The cohort is representative of the population of central Wisconsin. The dietary intake data will be a valuable resource for studies of gene-environment interactions. The Diet History Questionnaire should be followed up with periodic updates to assess changes in intake over time. The PMRP welcomes collaboration to enhance and expand gene-environment research.

## Competing interests

The authors declare that they have no competing interests.

## Authors' contributions

LS assisted in the interpretation of the data and drafted the manuscript. RB conducted the statistical analyses and assisted with data interpretation. DC assisted with data interpretation. WF collected the data. TK collected the data. LC assisted with study design and data interpretation. CAM was the Principal Investigator, contributing to all aspects of the project. All authors were involved in revising the manuscript and reviewed and approved the final version of the manuscript.
